# Bactericidal Activity of Silver Nanoparticles on Oral Biofilms Related to Patients with and without Periodontal Disease

**DOI:** 10.3390/jfb14060311

**Published:** 2023-06-02

**Authors:** Perla Alejandra Hernández-Venegas, Rita Elizabeth Martínez-Martínez, Erasto Armando Zaragoza-Contreras, Rubén Abraham Domínguez-Pérez, Simón Yobanny Reyes-López, Alejandro Donohue-Cornejo, Juan Carlos Cuevas-González, Nelly Molina-Frechero, León Francisco Espinosa-Cristóbal

**Affiliations:** 1 Chemical Biological Department, Institute of Biomedical Sciences, Autonomous University of Juarez City (UACJ), Envolvente del PRONAF and Estocolmo s/n, Ciudad Juárez 32310, Chihuahua, Mexico; al136416@alumnos.uacj.mx; 2 Master Program in Advanced Dentistry, Faculty of Dentistry, Autonomous University of San Luis Potosi, Manuel Nava Avenue, Universitary Campus, San Luis Potosí 78290, San Luis Potosi, Mexico; ritae_martinez@hotmail.com; 3 Department of Engineering and Materials Chemistry, Centro de Investigación en Materiales Avanzados, S. C., Miguel de Cervantes No. 120, Chihuahua 31109, Chihuahua, Mexico; armando.zaragoza@cimav.edu.mx; 4 Laboratory of Multidisciplinary Dental Research, Faculty of Medicine, Autonomous University of Queretaro, Clavel Street, Prados de La Capilla, Santiago de Querétaro 76176, Queretaro, Mexico; dominguez.ra@uaq.mx; 5 Institute of Biomedical Sciences, Autonomous University of Juarez City (UACJ), Envolvente del PRONAF and Estocolmo s/n, Ciudad Juárez 32310, Chihuahua, Mexico; simon.reyes@uacj.mx; 6 Master Program in Dental Sciences, Stomatology Department, Institute of Biomedical Sciences, Autonomous University of Juarez City (UACJ), Envolvente del PRONAF and Estocolmo s/n, Ciudad Juárez 32310, Chihuahua, Mexico; adonohue@uacj.mx (A.D.-C.); juan.cuevas@uacj.mx (J.C.C.-G.); 7 Division of Biological and Health Sciences, Autonomous Metropolitan University Xochimilco (UAM), Mexico City 04960, Mexico; nmolinaf@hotmail.com

**Keywords:** metal nanoparticles, silver, biofilms, periodontal diseases, anti-bacterial agents, humans

## Abstract

Background and Objectives: Periodontal disease (PD) is a multifactorial oral disease regularly caused by bacterial biofilms. Silver nanoparticles (AgNP) have offered good antimicrobial activity; moreover, there is no available scientific information related to their antimicrobial effects in biofilms from patients with PD. This study reports the bactericidal activity of AgNP against oral biofilms related to PD. Materials and Methods: AgNP of two average particle sizes were prepared and characterized. Sixty biofilms were collected from patients with (30 subjects) and without PD (30 subjects). Minimal inhibitory concentrations of AgNP were calculated and the distribution of bacterial species was defined by polymerase chain reaction. Results: Well-dispersed sizes of AgNP were obtained (5.4 ± 1.3 and 17.5 ± 3.4 nm) with an adequate electrical stability (−38.2 ± 5.8 and −32.6 ± 5.4 mV, respectively). AgNP showed antimicrobial activities for all oral samples; however, the smaller AgNP had significantly the most increased bactericidal effects (71.7 ± 39.1 µg/mL). The most resistant bacteria were found in biofilms from PD subjects (*p* < 0.05). *P. gingivalis, T. denticola,* and *T*. *forsythia* were present in all PD biofilms (100%). Conclusions: The AgNP showed efficient bactericidal properties as an alternative therapy for the control or progression of PD.

## 1. Introduction

Periodontal diseases (PD) are alterations that affect the supportive apparatus surrounding teeth, including gingival tissue, alveolar bone, periodontal ligament and cementum [1,2]. The PD involves inflammatory reactions induced basically by bacteria included in an oral biofilm in the periodontium [3,4], which is still a severe oral problem globally [3,5]. The initial stage of PD is called gingivitis, which is identified as an inflammation of the gingiva by the accumulation of bacteria or debris in a biofilm, which is a reactive and reversible condition [1,2]. However, periodontitis is presented in more severe stages of PD, which is a progressive periodontal condition, appearing as a chronic, destructive, irreversible inflammatory state [1,2,6]. The oral biofilm constituted by periodontal pathogens has been widely studied, determining that the interaction of specific species into the biofilm, the host response, and the development of PD, basically periodontitis, is a challenge [4,7]. The dysbiosis of the microbiota could alter bacterial ecology, facilitating the host’s inflammatory and immune response, ending in tissue destruction [8]. Among the bacteria that have been more directly involved in the pathogenesis of PD are *Actinobacillus actinomycetemcomitans* (*A. actinomycetemcomitans*), *Porphyromonas gingivalis* (*P. gingivalis)*, *Prevotella intermedia* (*P. intermedia*), *Bacteroides forsythus* (*B. forsythus*)*,* and *Treponema denticola* (*T. denticola*). Other bacterial species such as *Prevotella nigrescens* (*P. nigrescens*), *Campylobacter rectus* (*C. rectus*), *Peptostreptococcus micros* (*P. micros*), *Eikenella corroden* (*E. corroden*)*,* and *Fusobacterium nucleatum* (*F. nucleatum*) have a less relevant role, although they have occasionally been related to some forms of PD [4,7]. Particularly, the more prevalent bacteria in healthy oral biofilms could be mentioned, i.e., the Gram-positive bacteria such as *Streptococcus sanguinis* (*S. sanguinis*)*, Streptococcus oralis* (*S. oralis*)*, Streptococcus intermedius* (*S. intermedius*)*, Streptococcus gordonii* (*S. gordonii*)*, Peptostreptococcus micros* (*P. micros*)*, Gemella morbillorum* (*G. morbillorum*), and others. In contrast, the Gram-negative bacteria more frequently found are *Veillonella parvula, V. atypica, Capnocytophaga ochracea,* and others [4,7,9,10]. In periodontal disease biofilms, the microorganisms more frequently distributed in gingivitis belong to Gram-negative bacteria such as *Prevotella* spp., *Selenomonas* spp., and *Fusobacterium nucleatum* ss. *polymorphum*, among others. For a more severe conditions of PD, such as periodontitis, the main bacteria are classically described in the red-complex triad, including Gram-negative bacteria such as *T. denticola, P. gingivalis*, and *T. forsythia* [4,7,10,11].

The main treatments of PD are the control of bacterial growth immersed into the oral biofilm, divided into mechanical and chemical procedures [12]. The mechanical treatments of supra and subgingival biofilms are based on the control of bacterial growth proliferation, using mechanical instruments such as daily oral hygiene habits (cleaning devices, toothbrushes, dental floss, dental toothpicks, and others) [13,14] and specific professional therapeutics (surgical and non-surgical mechanical debridement) [12,15]. In the case of chemical therapeutics, some antimicrobial solutions have been demonstrated to help the action of mechanical procedures, permitting more effective antimicrobial controls against periodontopathogenic species [12]. The most common antimicrobial agents used for periodontal treatments are present in toothpaste and mouthwashes, which are accompanied, after mechanical debridement, by particular concentrations of the agent, according to the periodontal diagnosis and treatment [12,14]. Chlorhexidine gluconate (CHX) is the gold standard antimicrobial solution in the PD treatment used after surgical and non-surgical periodontal procedures, inhibiting bacterial proliferation consistently in the supra and subgingival dental sites during long-term periods, even in low contents [12,16,17]. Moreover, the solution of CHX has shown several adverse effects related to the pigmentation of hard dental tissues and tongue, mucosal irritation, taste disturbances, numbness and pain in mouth and tongue, xerostomia, and calculus, to name a few [18,19,20].

Even though CHX has been the best antimicrobial agent for treating PD, other alternatives have been studied. Some works have reported that antibiotics systemically administrated, such as amoxicillin/clavulanic acid, clindamycin, metronidazole, and the combination therapy metronidazole/amoxicillin, have demonstrated positive responses against various periodontal bacteria associated with destructive PD [21,22]. However, at the same time, the increase in antimicrobial resistance is a significant limitation in long-term administration [22,23], including periodontal microbial affections [24]. In this sense, it is necessary to explore new and more effective antimicrobial agents [25,26,27] with properties that improve periodontal therapy through bacterial growth inhibition and the reduction of antibiotic resistance. The literature has recommended the use of silver nanoparticles (AgNP) for the control of bacterial proliferation, due to their excellent bacteriostatic and bactericidal properties in many microbial species, including oral microorganisms [28,29,30,31,32,33]. A significant number of researchers report the evaluation of the bactericidal effect of the AgNP against specific bacterial species using standard bacterial stocks provided by Microbial Type Culture Collection (MTCC) [34] or American Type Culture Collections (ATCC) [35,36] catalogs. Therefore, there are limited investigations that have determined the antimicrobial activity of AgNP using exclusively clinical bacterial oral biofilms, focused specifically on individual periodontal pathogens [34,37,38,39]. The purpose of this study was to evaluate the antimicrobial activity of AgNP against several oral biofilms isolated from patients with and without PD and to explore the associations of the antimicrobial activity of AgNP with the sociodemographic and clinical characteristics of the subjects under study. The results of this study will contribute both to a better understanding and the safe use of these metallic nanomaterials as an alternative approach for the prevention and control of PD.

## 2. Materials and Methods

### 2.1. Materials

Silver nitrate (AgNO_3_, CTR Scientific, Monterrey, Nuevo León, Mexico), gallic acid (C_7_H_6_O_5_, Sigma Aldrich, Saint Louis, MO, USA), sodium hydroxide (NaOH, Jalmek Scientific, San Nicolás de los Garza, Mexico), ammonia hydroxide (NH_4_OH, Jalmek Scientific), Müller-Hinton broth (MH, BD™ Difco™, Rockville, MD, USA), 2.0% chlorhexidine gluconate (Consepsis, Ultradent Products Inc, South Jordan, UT, USA), were used and stored according to manufacturer’s recommendations.

### 2.2. Preparation and Characterization of AgNP

AgNP of two average particle sizes were prepared using the synthesis method previously reported [40]. First, 0.169 g of silver nitrate (AgNO_3_, CTR Scientific, Monterrey, Mexico), as a precursor agent, was dissolved in 100 mL of deionized water with magnetic stirring. Afterward, 10 mL of deionized water with 0.1 g of gallic acid (C_7_H_6_O_5_, Sigma Aldrich, St. Louis, MI, USA), used as a reducing agent, was immediately added to the first solution. Finally, the pH was adjusted to 11 with a 1 M solution of sodium hydroxide (NaOH, Jalmek Scientific, San Nicolás de los Garza, Mexico) for the particle size stabilization. For the second particle size, 10 mL of deionized water with 0.5 g of gallic acid was incorporated into AgNO_3_ solution, prepared as mentioned above, adjusting the pH to 11 using a solution of ammonium hydroxide (NH_4_OH, Jalmek Scientific, San Nicolás de los Garza, Mexico) under magnetic stirring. Both solutions were stirred for 10 min under laboratory conditions. The characterization of the two samples of AgNP was carried out by dynamic light scattering (DLS) for the determination of average particle size and particle size distribution. The zeta potential of particles was analyzed using a nanoparticle analyzer (DLS, Nanoparticle Analyzer, Nano Partica SZ-100 series, HORIBA Scientific Ltd., Irvine, CA, USA), while transmission electron microscopy (TEM, Phillips CM-200) was used for the determination of particle shape using a voltage accelerating of 25 kV.

### 2.3. Patient Recruitment

A consecutive nonprobabilistic sampling was carried out to select patients from the Dental Admission Clinic belonging to the Stomatology Department at the Autonomous University of Ciudad Juárez (UACJ), Mexico. All recruited patients voluntarily signed an informed consent before taking the clinical samples regarding the ethical guidelines of the Helsinki Declaration (2008). The study was approved by the Biomedical Sciences Institute Research Committee (ICB), UACJ (project ID RIPI2019ICB5). The study involved 60 subjects between 30 to 50 years old, who were divided into two groups: (a) 30 subjects with PD and (b) 30 subjects without PD (healthy). The presence of PD was defined by evident partial or total gingival inflammation, with induced or spontaneous blood bleeding, up to the presence of apical migration of the periodontal attachment tissue in at least one single tooth during the oral examination. Clinical intern experts of the Dental Social Services of the Dentistry program at ICB-UACJ diagnosed the PD.

### 2.4. Sampling of Oral Biofilms

The oral biofilms were collected from subgingival and supragingival oral sites from the teeth of patients with or without PD, using a sterile wooden toothpick to create a mechanical scraping in the interproximal, vestibular, lingual, or palatal surface in a single direction. The toothpicks with the biofilm samples were immediately placed in a tube containing 5 mL of Müller–Hinton broth (MH, BD™ Difco™, Rockville, MD, USA), and incubated for anaerobic bacteria at 37 °C for 24 h.

### 2.5. Initial Bacterial Growth and Standard Microbial Suspension

The initial bacterial growth was measured using the optical density (OD) for each oral biofilm before antimicrobial activity tests. Once microbiological samples were incubated, 100 µL of each bacterial sample were added to 3 mL of phosphate buffer solution (PBS). The absorbance level was analyzed using a spectrophotometer (Eppendorf, BioPhotometer Plus, München, Germany) at a wavelength of 550 nm by triplicate. Then, the concentration of microbial samples was standardized at 1.3 × 10^8^ colony-forming units per milliliter (CFU/mL), obtained when the bacterial suspensions reached an absorption of 0.126 at 550 nm of wavelength, according to the McFarland scale. Finally, all bacterial suspensions were diluted with PBS and homogenized in a concentration of 1.3 × 10^6^ CFU/mL, which was used for all microbiological tests.

### 2.6. Antimicrobial Test

The antimicrobial assay was carried out according to the method previously reported [41]. The minimal inhibitory concentration (MIC) of AgNP, through microdilution plates with 96 wells, was used to determine the antimicrobial activity. From the second column to the twelfth, 100 µL of MH broth was placed in each well. After that, 200 mL of each antimicrobial solution was placed in the first column. Then, serial dilutions in a 1:1 proportion were made up to column eleven. Thus, 100 µL of the standardized bacterial suspensions (1.3 × 10^6^ CFU/mL) were added to all wells. Finally, each plate was incubated in anaerobic conditions for 24 h at 37 °C. The MIC values were identified in the last well with no bacterial growth determined by visual and stereomicroscopic comparisons using turbidity parameters. This procedure was carried out for each oral biofilm related to PD and healthy (no PD) subjects in triplicate. Columns one and twelve were assigned as positive (no bacterial growth) and negative (bacterial growth) controls, respectively. The CHX solutions were identified as a gold antimicrobial standard reference.

### 2.7. Identification of Bacteria by Polymerase Chain Reaction (PCR)

Six samples of oral biofilms from patients with and without PD were randomly selected to identify periodontal bacteria using a polymerase chain reaction (PCR) assay. The presence of *P. gingivalis*, *T. forsythia*, *T. denticola*, *P. intermedia*, *F. nucleatum*, and *A. actinomycetemcomitans* was determined according to previously reported methods [42,43]. Specific primers for the detection of periodontal bacterial species were used according to previous methods by Tran and Rudney [44], Stubbs et al. [45], Watanabe and Frommel [46], Ashimoto et al. [47], and Poulsen et al. [48]. Positive and negative controls were included in each PCR set. All PCR products were submitted to electrophoresis in 2% agarose gels, stained with ethidium bromide, and analyzed under UV light (E-Gel Imager System with UV Base, Thermo Fisher Scientific, Life Technologies, Waltham, MA, USA).

### 2.8. Statistical Analysis

The general distribution of patients with and without PD, according to gender and bacterial identifications by PCR, was expressed in frequencies and percentages. The homogeneity of the study groups was examined using Pearson´s chi-square test. The values of age from patients, OD of initial bacterial growth, and MIC values from the antimicrobial activity of treatments were presented in means and standard deviations. The normality of variables was analyzed using the Shapiro–Wilks test. The independent comparisons according to treatments, gender, and type of oral biofilm were calculated using the Mann–Whitney U statistical test for nonparametric variables. In addition, Spearman´s rho analysis was used to identify correlations of age among OD, and MIC values from patients with and without PD. All statistical analyses were performed using IBM-SPSS software (SPSS, version 25, Chicago, CA, USA) considering statistical significance when *p* < 0.05.

## 3. Results

### 3.1. Characterization of AgNP

**Figure 1:**
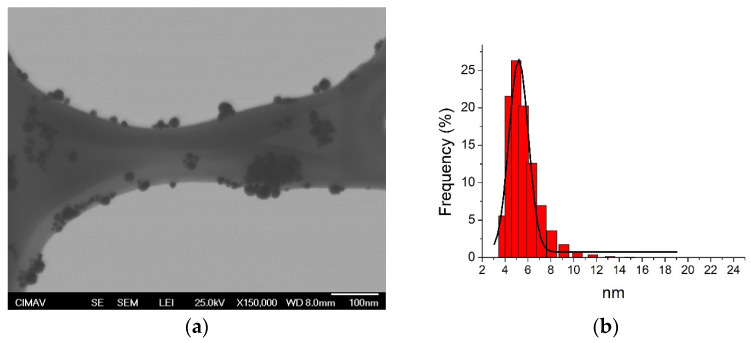

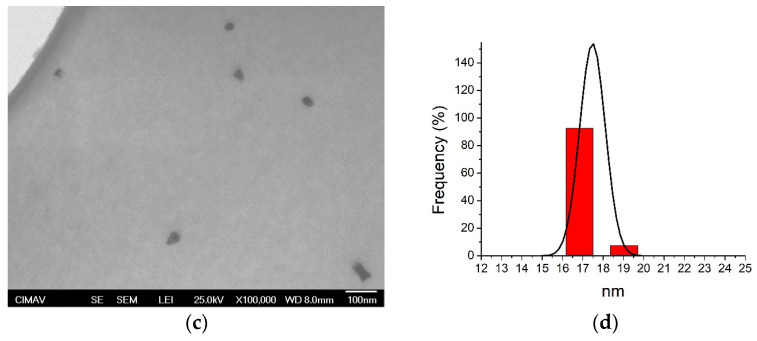
Transmission electron microscopy (TEM) and dynamic light scattering (DLS) analysis of silver nanoparticles (AgNP). (**a**,**b**) 5.4 nm; (**c**,**d**) 17.5 nm.

Table 1 reports the physical characteristics of AgNP determined by DLS and TEM. In the DLS analysis, single peaks and well-defined sizes were determined from the two colloidal solutions of AgNP (5.4 ± 1.3 and 17.5 ± 3.4 nm), which indicates that both samples of Ag had a narrow and uniform particle size distribution (Figure 1b,d), identifying spherical shapes for smaller and larger Ag particles, respectively (Figure 1a,c). The zeta potential results showed negative electrical charge values for smaller and larger sizes, with different electrical intensities that suggest good electrical stability (−38.2 ± 5.8 and −32.6 ± 5.4 mV, respectively), preventing particle agglomeration. These results support that the particles of both families have adequate size distribution and good superficial electrical properties.

### 3.2. Distribution of Patients

Table 2 shows the general distribution of patients in the two study groups. In general, the subjects included in the PD and healthy groups were young adult patients. The subjects with PD (39 ± 6.9 years old), including women (37 ± 7.7 years old) and men (40.5 ± 5.8 years old), were older compared to the total of healthy patients (28 ± 8.9 years old) and both genders (28.3 ± 9.4 and 27.5 ± 8.6 years old, respectively); however, no statistical differences were identified (*p* > 0.05). Additionally, the subjects included in the PD and healthy groups had similar distribution (47% for women and 53% for men), determining no significant differences between both groups according to gender (*p* > 0.05). Those results suggest that the age and gender of patients had a similar distribution in the PD and healthy groups.

### 3.3. Bacterial Growth of Biofilms

**Figure 2:**
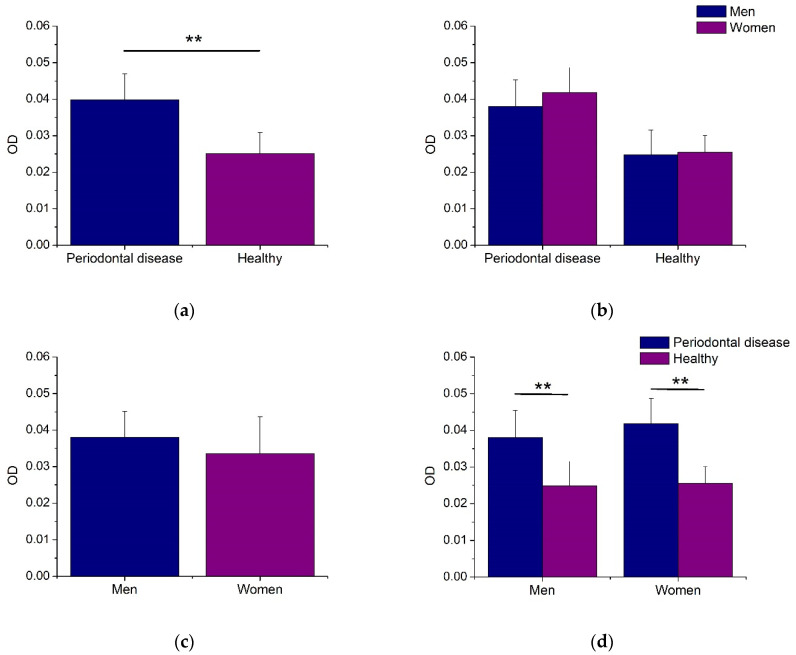
Initial growth of biofilm samples from subjects with and without periodontal disease. All values are expressed in mean and standard deviation. Asterisks indicate statistical differences (*p* < 0.01).

Figure 2 illustrates the initial bacterial growth for each biofilm sample. As noted, the oral biofilms from men and women subjects with PD showed statistically increased bacterial growth compared to healthy patients (Figure 2a,d). Therefore, the biofilms from women and men patients had similar bacterial growth activity, with no statistical differences (Figure 2b,c). These results indicate that the bacterial growth capacity of microorganisms included in the oral biofilms is associated with the presence of PD.

### 3.4. Antimicrobial Activity of AgNP

**Figure 3:**
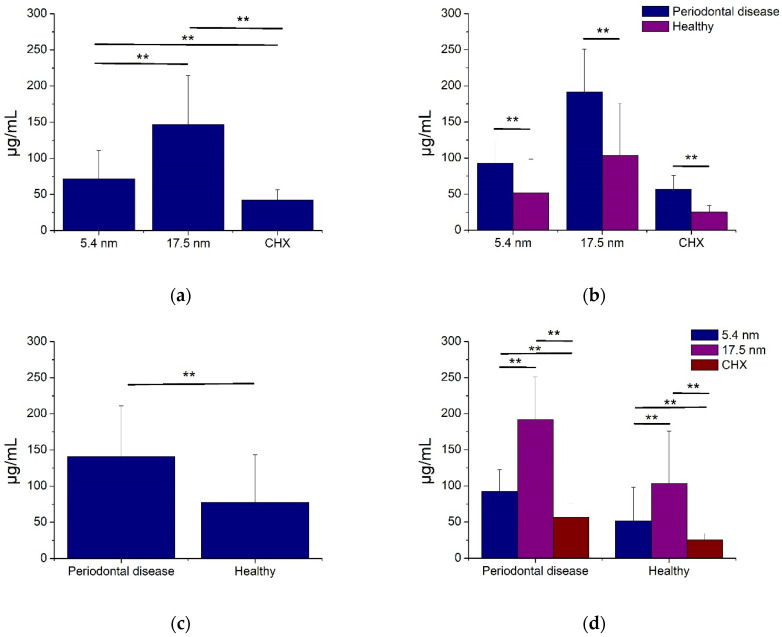
Antimicrobial activity of AgNP against biofilms associated with periodontal disease. All values are expressed in mean and standard deviation. Asterisks indicate statistical differences (*p* < 0.01).

**Figure 4:**
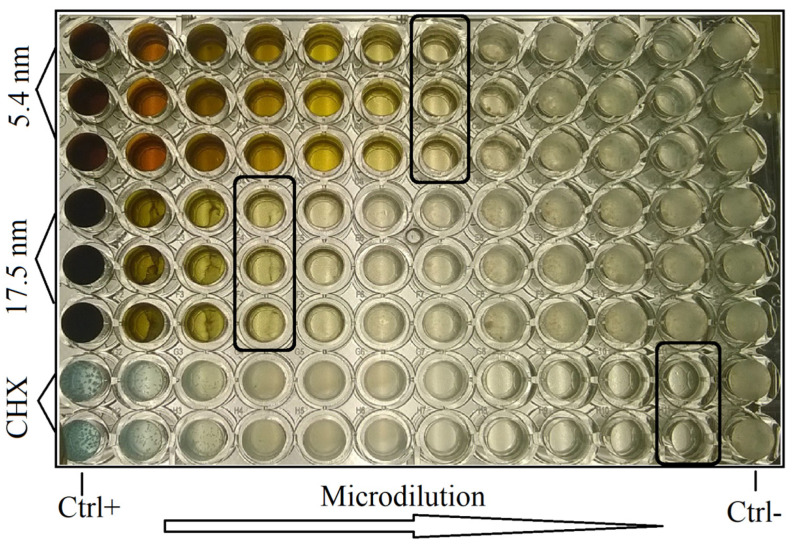
Representative microdilution plate with MIC values of AgNP and CHX in oral biofilm from PD subject. Black squares represent MIC values.

Figure 3 and Figure 4 show the antimicrobial activity of AgNP in oral biofilms of periodontal disease and healthy patients. As seen, smaller (5.4 nm) and larger (17.5 nm) AgNP, as well as CHX, had good bacterial inhibition activity for all oral biofilms from PD and healthy subjects (Figure 3 and Figure 4). The smaller AgNP showed significantly better antimicrobial activity (71.7 ± 39.1 µg/mL) compared to larger particles (146.9 ± 67.3 µg/mL). However, the CHX had the highest bacterial inhibition of dental biofilms (Figure 3a), even for oral biofilms from PD and healthy subjects (Figure 3d). Moreover, the microorganisms involved in periodontal disease samples demonstrated, statistically, more resistance activity (141.0 ± 69.9 µg/mL) compared to non-PD biofilms (77.7 ± 65.5 µg/mL) for any antimicrobial solution (Figure 3c), including CHX (Figure 3b). These results suggest that both families of AgNP had good antibacterial activity in all samples of oral biofilms from patients with and without PD, associating this activity with the particle size and the type of oral biofilm.

**Figure 5:**
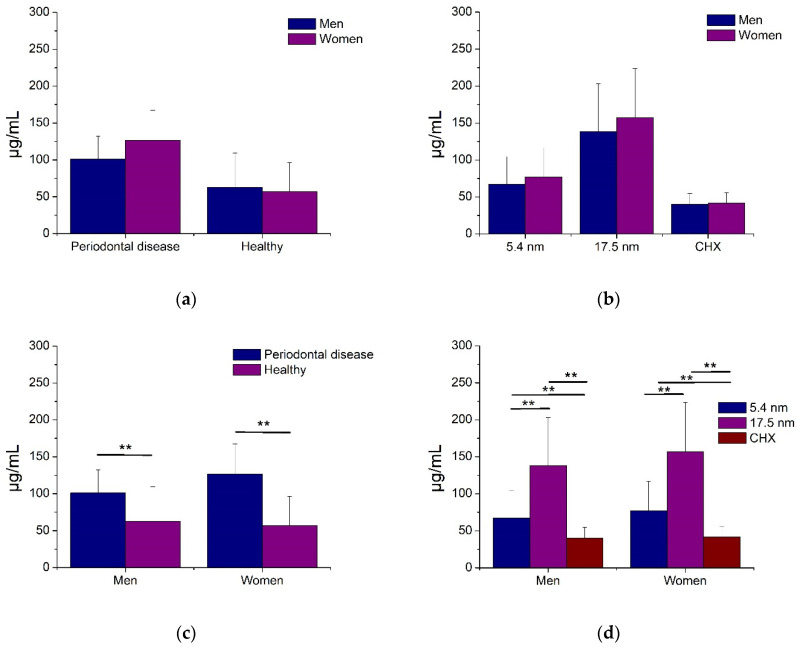
Antimicrobial activity of AgNP against biofilms associated with periodontal disease and gender. All values are expressed in mean and standard deviation. Asterisks indicate statistical differences (*p* < 0.01).

Figure 5 shows the antimicrobial activity of AgNP in oral biofilms from patients with and without PD according to gender. Women showed a tendency to higher resistance to any AgNP sample or CHX solution compared to men; consequently, no significant differences were determined (Figure 5a,b). On the other hand, the oral biofilms from periodontal disease patients and smaller AgNP (5.4 nm) demonstrated, statistically, that for both genders, there was more increased bactericidal activity than biofilms from healthy patients and larger Ag particles, respectively (Figure 5c,d). For all cases, the CHX solution had the best antimicrobial effects (Figure 5d). These results illustrate that gender is not associated with the antimicrobial effect of AgNP, acting similarly for oral biofilm from women and men patients.

The Spearman correlation results of OD and MIC, according to age from oral biofilms of patients with and without PD, are summarized in Table 3. In general, positive correlations were identified at the initial growth of biofilms (*rho* = 0.501), smaller (*rho* = 0.223), and larger (*rho* = 0.223) Ag particles for both groups (periodontal disease and healthy samples). Thus, the initial bacterial growth demonstrated only a significant correlation among the age of patients (*p* < 0.05). Furthermore, specific positive correlations were determined at the initial growth of oral biofilms from periodontal disease (*rho* = 0.179) and non-periodontal (*rho* = 0.087) disease patients, identifying no significant correlations (*p* > 0.05). Although PD biofilms showed positive correlations for smaller (*rho* = 0.021) and larger (*rho* = 0.122) AgNP, negative correlations for healthy biofilm samples were also determined for both particle families (*rho* = −0.248 and −0.042, respectively); however, no statistical correlations were found for any particle sample (*p* > 0.05). These results suggest that the initial bacterial growth capacity of both oral biofilms increases gradually with age (*p* < 0.05), demonstrating a particularly high tendency for periodontal disease biofilms. On the other hand, the concentration of AgNP for both particle sizes acts similarly at any age. However, the PD biofilms had a trend to need higher contents of AgNP according to age, while biofilms from patients with no PD showed an opposite tendency, requiring gradually lower concentrations of nanoparticles with respect to age.

### 3.5. Distribution of Periodontal Bacteria by PCR Assay

Periodontal bacteria profiles, identified by PCR, are shown in Table 4. As observed, the distribution of the population was represented by young adult patients with ages from 31 to 35 years old (32.5 ± 1.6 years old), in which females were more frequent (66.7%) compared to male subjects (33.3%). The identification of periodontal bacteria was represented mainly by *P. intermedia* (100%) and *F. nucleatum* (100%), followed by *P. gingivalis* (83.3%), *T. denticola* (83.3%), T. *forsythia* (66.7%) and, finally, *A. actinomycetemcomitans* (16.7%). Notably, the *P. gingivalis, T. denticola,* and *T*. *forsythia* strains were present in all oral biofilms from subjects with PD (100%). In contrast, the patients with no PD had less frequency of these bacteria in the biofilms (66.7, 66.7, and 33.3%, respectively). The *A. actinomycetemcomitans* was only present in one oral biofilm from subjects with PD (33.3%).

## 4. Discussion

This study identified that the AgNP significantly inhibits the bacterial growth of oral biofilms related to PD and healthy patients. The particle size and specific oral biofilms were associated with intervening in the antimicrobial activity of AgNP. Although gender tended to offer more bacterial resistance to both AgNP families and CHX solution, particularly for women, no significant associations were found. Additionally, significant correlations were located for the initial bacterial growth activity for PD and healthy oral biofilms, indicating that the growth of microorganisms involved for each biofilm was directly proportional to the age of the subjects. Although there was a tendency for PD biofilms to increase their antimicrobial resistance to both AgNP samples, no significant correlations were determined, suggesting that smaller and larger AgNP had statistically similar bacterial inhibition capacities for any age. To the best of our knowledge, this is the first study that evaluated the antimicrobial activity of two sizes of AgNP against representative oral biofilm samples taken from patients with and without active PD. Those results offer more precise and reliable information about the bactericidal effects of AgNP and oral biofilms obtained directly from patients, where complex microbiological interactions in different bacterial species with particular metabolic and microbiological characteristics were involved.

Various studies have reported synthesis methods for the preparation of AgNP using similar characteristics to this work, obtaining almost similar sizes and shapes of AgNP [31,41]. In this study, the synthesis of the AgNP included silver nitrate as a precursor and gallic acid as a reducing and stabilizing agent, which promoted the oxidation reaction of the phenol groups, facilitating the reduction of the silver ions [49]. The reaction was carried out at pH 10 for 17.5 nm and at pH 11 for 5.4 nm. At these pHs, the reaction is very fast and allows spherical morphology [31,50]. In the synthesis of the larger AgNPs, ammonium hydroxide caused faster reaction kinetics, having a promotion of hydrolysis, favoring the formation of particles of larger size [51] with more stable sizes [50,52]. As for 5.4 nm AgNP, the addition of sodium hydroxide promoted a sudden temperature gradient that resulted in rapid nucleation, thus inhibiting the growth of the particles [53,54] and limiting the particle agglomeration associated with the electrical charge [41,55]. Despite those reports, some variations might occur during the synthesis process. The results from this study showed that smaller and larger AgNP had narrow sizes with low standard deviations, which suggests adequate reproducibility of the synthesis technique for both particle sizes. Additionally, it is known that specific intervals of electrical charge particle surface of AgNP (from +31 to −30 mV) and high pH (>7) promote better conditions in colloid Ag dispersions to prevent particle agglomeration [30,56,57,58]. The zeta potential values support adequate electrical charges on the particle surface, facilitating the monodispersing and preventing particle agglomeration due to well-defined and distributed electrical surface energies on the Ag particles (−38.2 ± 5.8 mV for smaller and −32.6 ± 5.4 mV for larger particles). Results from the physical characterization of the particles indicate the improvement of the bactericidal activity of both AgNP samples, maintaining a colloid with more monodisperse particle dispersion, increasing the surface area and contact, limiting the agglomeration activity, resulting in more efficient bacterial inhibition behaviors against the oral biofilms [56,58].

The literature reports that the AgNP showed significantly higher bacteriostatic and bactericidal effects against five oral pathogenic microorganisms (*S. mutans*, *S. oralis*, *L. acidophilus*, *L. fermentum*, and *C. albicans*) compared to CHX, at less than five-fold concentration [34]. Additionally, another work reported that the AgNP had effective antibacterial activity against different microbial strains, including *P. gingivalis* and *Enterococcus faecalis*, which are bacterial species that have been strongly associated with PD and drug-resistant endodontic infectious, respectively [37]. Other works used various types of AgNP to evaluate the antimicrobial effect, using glutathione-stabilized AgNP [38] and AgNPs synthesized with *Ocimum Sanctum* leaf extract [59] against different oral pathogens associated with dental caries (*S. mutans*) and PD (*F. nucleatum*), particularly periodontitis (*P. gingivalis, P. intermedia*, and *A. actinomycetemcomitens*). It was determined that those AgNP exerted strong antibacterial functions in a concentration-dependent manner on the different microbial strains, suggesting the application of these nanomaterials for antibacterial treatments for the prevention of dental caries, periodontal conditions, or any other dental application related to oral biofilms [38,59]. A more recent study evaluated the antimicrobial and anti-inflammatory responses of polymeric PVA/chitosan thin films, containing AgNP and ibuprofen for the treatment of PD against Gram-positive (*Staphylococcus aureus*, ATCC 25, 923) and negative (*Pseudomonas aeruginosa* ATCC 27, 853; *Klebsiella pneumoniae* ATCC BAA-1705; and *P. gingivalis* ATCC 33, 377) bacteria. This resulted in biocomposite films with good bactericidal and biocompatible properties in representative oral pathogens, including microbial strains associated with PD (*P. gingivalis*) [39]. Our results agree with those previously reported, suggesting the great bactericidal effectiveness of AgNP against all bacterial biofilms from patients with periodontal and non-PD, determining better antimicrobial activities for smaller AgNP (Figure 3a,d) with significant microbial resistance to AgNP from microorganisms included in oral biofilms of patients with PD biofilms (Figure 3b,c). The evaluation of initial bacterial growth was made to determine the bacterial growth rate before the antimicrobial assay and defined differences in proliferation speed between different types of biofilms. This exploration determined differences among metabolic characteristics from various biofilms. We used multispecies cell suspensions initially from clinical oral biofilms, in which the presence of particular microbial species was confirmed by the PCR assay. Although the results from correlations indicated that the microorganisms included particularly in PD biofilms had more predispositions to accelerate the initial bacterial growth and, although no significant correlations were identified among AgNP samples, a strong tendency to resist the antimicrobial activity of the nanoparticles was determined (Table 3). The difference in the inhibitory effect between both sizes of AgNP was possibly due to their physical characteristics, which are observed in Table 1 and Figure 1. These findings suggest that the inhibitory effect of AgNP is inversely proportional to the size of the nanoparticle, with relevant participation of the PD biofilms to create more difficult conditions to facilitate the adequate bactericidal activities of AgNP.

Additionally, the female gender plays an important role in limiting the antimicrobial action of these nanostructured materials. In this sense, the small size of the AgNP can probably increase the ratio between the surface area and the volume in a very important way, which leads to significant modifications in their physical, chemical, and biological properties, including potentiated bacterial inhibitory effects [60,61]. The fact that the inhibitory effect with both sizes of AgNP was lower in dental plaque samples from patients with PD is possibly due to the bacterial species present in dental plaque. In the etiology of PD, there is not a single bacterial species involved. Rather, it is considered a polymicrobial infection, in which various microorganisms are involved, either in combination in the same period or in a sequential way [4,62,63]. Some investigations support the existence of the microbiota resistant to conventional antimicrobials in the dental plaque of patients with PD [64,65]. This capacity of the bacteria could explain the decrease in the inhibitory effect of AgNP mainly in biofilms associated with PD bacteria.

It is well known that in oral biofilms from patients without PD, a lower bacterial number exists than PD in subgingival biofilms, due to the control of bacterial growth and waste sub-products derived from metabolic activities in oral biofilms during regular oral hygiene habits [4,7,10]. Some authors have previously reported that in the oral dental plaque in patients with PD, the number of bacteria is greater [66]. This indicates that the first microbial colonizers adhere to the acquired film by specific molecules called adhesins, which are present on the bacterial surface [67]. Through the proliferation of attached species, the colonization and growth of other bacteria can also occur. In this microbiological succession, there is a transition from an aerobic environment, characterized by facultative microorganisms, to an anaerobic one, due to the consumption of oxygen by those first colonizers that favors the predominance of anaerobic microorganisms [66,67]. This information suggests that the antimicrobial resistance of oral biofilms from subjects with PD conditions are strongly associated with the specific distribution and type of microbial species in the oral biofilms, which is, at the same time, related to the oral hygiene habits of each patient and not only to the physical and chemical properties of AgNP.

On the other hand, immunological investigations have also reported that the innate immune response is more regulated in women than men, which limits the presence and progression of PD by the control of the growth of bacterial pathogens [68]. Additionally, the presence of estrogens (female hormone related to the reproductive system) increases gamma interferon (INF-γ) production, which represents an important role in the development of PD, principally periodontitis [69], suggesting an association with bone resorption. However, authors have also reported opposite approaches through in vitro and in vivo studies about sexual dimorphisms in oral bacterial infectious related to alveolar bone loss, resulting in increased inflammatory responses in males, which might create better biochemical conditions for the presence of more severe PD stages in comparison to females [70]. Although the relationship between gender and PD is not still clear, some studies have revealed interesting predispositions, according to gender, to increase the presence and severity of PD, including more antimicrobial resistance to certain microbial agents. Consequently, such specific hormonal factors, identified exclusively in women subjects, could alter the immune response in the PD, leading to the homeostasis loss from periodontal tissues and facilitating the development of gingivitis and periodontitis [71,72]. Results from this research regarding the characteristics of female patients show that there is a need for a greater concentration of AgNP and CHX treatments compared to males and, although no significant differences were found according to gender, an interesting tendency among females to be more resistant to all antimicrobial agents was identified. A possible explanation is that the concentrations of AgNP and CHX solutions needed to generate antimicrobial activity in women patients were greater than in men due to certain physiological and metabolic stages that can alter the hormonal conditions exclusively for women, such as puberty, menstrual cycle, pregnancy, menopause, postmenopause, and the use of contraceptive agents, promoting the modification of the immune response to periodontal disease, which leads to the homeostasis loss of periodontium tissue, facilitating the development of gingivitis and periodontitis [72,73,74].

The action mechanism of AgNP is still a controversial issue, but it is suggested that AgNP might gradually release silver ions that inhibit the production of adenosine triphosphate (ATP) and deoxyribonucleic acid (DNA) replication, which are fundamental elements for cell survival, but at the same time, promote the production of reactive oxygen species (ROS) and, subsequently, cell death [75,76,77]. This effect could be explained because silver ions have a strong affinity for the electron-donating groups present in various bacterial cells that contain sulfur, oxygen, or nitrogen [78], facilitating internalized particles into the bacterium due to their narrow size [79,80,81]. Then, AgNP releases silver ions and simultaneously joins to sulfhydryl groups of biomolecules and with phosphorosulfur compounds such as DNA, inactivating and altering the bacteria and cytoplasm leading to cell death [75,76,77]. In this sense, the action mechanism of AgNP might synergistically involve particular physical and chemical conditions such as silver release, chemical affinity with the cell wall, shape, size, electrical charge on the surface, concentration, distribution, and others [75,81,82]. Yet, specific microbiological and sociodemographic characteristics related to biofilms and patients such as type of oral infectious disease, particular microbial species, drug-resistance bacteria, type of cell wall, gender, oral hygiene habits, systemic disease, genetic, age, as well as immunological and metabolic conditions, among others, may be factors as well [41,73,83,84].

Additionally, the potential damage offered by nanomaterials, exclusively AgNP, to organs and systems of the body has been considered to promote the safe use of these metallic nanomaterials in biomedical uses. Studies have reported that AgNP can be distributed to specific organs according to a wide variety of routes, including inhalation, ingestion, skin contact, subcutaneous, or intravenous injection [85]. These routes will determine the specific organs where the AgNP will penetrate and accumulate. The absorbed AgNP are distributed in various organs and tissues such as the spleen, liver, stomach, lung, heart, brain, small intestine, and muscles, even in teeth and bones [86,87]; promoting cytotoxicity in dermal, ocular, respiratory, hepatobiliary, neural, or reproductive systems, limiting the application of these nanomaterials [86,87,88]. In addition, experiments have revealed that cytotoxicity is related to the chemical transformation of AgNP into silver ions (Ag+), facilitating the induction of cellular biochemical changes [89]. On the other hand, investigations have reported that AgNP could play an important anti-inflammatory role to reduce wound inflammation, modulation of fibrogenic and proinflammatory cytokines, and apoptosis in inflammatory cells [90]. Moreover, various studies have determined that in specific conditions, AgNP did not produce significant hepatoxicity or immunotoxicity during in vivo studies with rats [87,91]. Additionally, an in vivo human oral time-exposure study reported that the commercial nanoscale silver particle solutions administrated to humans did not promote clinically important changes in the metabolic, hematologic, urine, or physical findings [92]. This means that it is very necessary to investigate the toxicity mechanisms of AgNP to elucidate the potential cytotoxicity, long-term adverse effects, routes of administration, doses, and other physical and biochemical properties to determine a well-defined and safe therapeutic with AgNP.

Although this study determined that AgNP exerts an inhibitory effect on oral biofilms in patients with PD, it is necessary to identify more realistic conditions from antimicrobial evaluations in future investigations, thus determining the antimicrobial particularities of AgNP among specific and well-controlled bacterial distributions from oral biofilms. This perspective will permit us to understand the relationship between the antimicrobial effects of AgNP related to particular microbiological conditions of biofilms, as well as to clinical and sociodemographic conditions from sampled patients such as oral or systemic disease, medical conditions, oral hygiene or eating habits, pharmacological treatment, race, genetics, gender, age, or any other particular conditions derived from patients. This will undoubtedly generate a better understanding, allowing for the recommendation of the safe use of AgNP as a potential antimicrobial alternative to control the presence and severity of PD in humans.

## 5. Conclusions

AgNP exerted a statistically significant growth inhibitory effect against clinically isolated oral biofilms from patients with and without PD. The primary antimicrobial associations of AgNP were determined according to particle size from AgNP and type of oral biofilm, determining a higher inhibitory effect for the smaller particles, while the most resistant antimicrobial activity was presented for microorganisms involved in PD biofilms. On the other hand, although no significant differences among the antimicrobial activity of AgNP were defined according to gender, an interesting tendency was shown for female subjects, having the predisposition to demonstrate a more resistant bactericidal activity to AgNP compared to males. Additionally, significant correlations were found among the initial bacterial growth, in particular, a trend for the PD group to increase the growth capacity, while the antimicrobial activity of both AgNP samples acted similarly in any age for any oral biofilm. Despite the fact that AgNP showed latent antimicrobial properties suggesting its potential for application as a complementary therapy for the prevention and control of PD, new investigations on AgNP using novel methodological approaches with more clinical and sociodemographic information from clinical oral biofilms, as well as other oral biofilms related to infectious diseases in the oral cavity, are strongly recommended.

## Figures and Tables

**Table 1:** Physical characteristics of AgNP families.

AgNP	DLS (nm)	Shape	Concentration (µg/mL)	Zeta Potential (mV)
5.4 nm	5.4 ± 1.3	Spherical	1070	−38.2 ± 5.8
17.5 nm	17.5 ± 3.4	Spherical	1070	−32.6 ± 5.4

DLS = dynamic light scattering. DLS and zeta potential results are expressed in mean, standard, and zeta deviation, respectively.

**Table 2:** General sociodemographic distribution of study groups.

	Periodontal Disease *n* = 30 Subjects (%)	Control (Healthy) *n* = 30 Subjects (%)
Age (years old)	39 ± 6.9	28 ± 8.9
Women	37 ± 7.7	28.3 ± 9.4
Men	40.5 ± 5.8	27.5 ± 8.6
Gender		
Women	14 (47)	14 (47)
Men	16 (53)	16 (53)

Values from age are expressed in mean and standard deviation. There were no statistical differences between periodontal disease and healthy patients according to age and gender (*p* > 0.05).

**Table 3:** Spearman’s rho correlation of OD and MIC, according to age from periodontal disease and healthy patients.

Variable	Periodontal Disease (*r*)	*p*-Value	Healthy (*r*)	*p*-Value	Total (*r*)	*p*-Value
OD	0.179	0.345	0.087	0.647	0.501	0.000 **
AgNP 5.4 nm	0.021	0.914	−0.248	0.186	0.223	0.087
AgNP 17.5 nm	0.122	0.521	−0.042	0.825	0.223	0.074

** indicate significant correlations (*p* < 0.01).

**Table 4:** Periodontal bacterial profiles detected by PCR.

Groups	No Periodontal Disease			Periodontal Disease		
Subjects	1	2	3	4	5	6
Age (years)	32	31	34	32	35	31
Gender	F	M	F	F	M	F
Bacterial strains						
*P.gingivalis*	+	−	+	+	+	+
*T. forsythia*	−	−	+	+	+	+
*T. denticola*	+	−	+	+	+	+
*P. intermedia*	+	+	+	+	+	+
*F. nucleatum*	+	+	+	+	+	+
*A. actinomycetemcomitans*	−	−	−	−	−	+

PCR: polymerase chain reaction. Gender is expressed in female (F) and male (M); + indicates the presence of bacteria; − indicates the absence of bacteria.

## Data Availability

All data obtained from this study can be found in the research archives of the Master’s Program in Dental Sciences of the Autonomous University of Ciudad Juarez and can be requested through the corresponding author.
